# Serum Magnesium Is Associated with Long-Term Survival of Non-ST-Elevation Myocardial Infarction Patients

**DOI:** 10.3390/nu15194299

**Published:** 2023-10-09

**Authors:** Amitai Segev, Michael Shechter, Avishai M. Tsur, David Belkin, Hofit Cohen, Amir Sharon, Nira Koren Morag, Ehud Grossman, Elad Maor

**Affiliations:** 1 The Leviev Cardiothoracic & Vascular Center, Sheba Medical Center, Ramat Gan 5262504, Israel; shechtes@netvision.net.il (M.S.); eladmaor@gmail.com (E.M.); 2 The Faculty of Medicine, Tel Aviv University, Tel Aviv 6997801, Israelbelkin@mail.tau.ac.il (D.B.); hofit.cohen@sheba.health.gov.il (H.C.); ehud.grossman@sheba.health.gov.il (E.G.); 3 Department of Medicine, Sheba Medical Center, Ramat Gan 5262504, Israel; 4 Israel Defense Forces, Medical Corps, Ramat Gan 5262504, Israel; 5 The Bert W. Strassburger Lipid Center, Sheba Medical Center, Ramat Gan 5262504, Israel

**Keywords:** NSTEMI, magnesium, endothelial dysfunction, prognosis, mortality

## Abstract

Background: Low serum magnesium (sMg) is associated with cardiovascular risk factors and atherosclerotic disease. Objective: To evaluate the association between sMg levels on admission and clinical outcomes in hospitalized non-ST-elevation myocardial infarction (NSTEMI) patients. Methods: A retrospective analysis of all patients admitted to a single tertiary center with a primary diagnosis of NSTEMI. Patients with advanced chronic kidney disease were excluded. Clinical data were collected and compared between lower sMg quartile patients (Q1; sMg < 1.9 mg/dL) and all other patients (Q2–Q4; sMg ≥ 1.9 mg/dL). Results: The study cohort included 4552 patients (70% male, median age 69 [IQR 59–79]) who were followed for a median of 4.4 (IQR 2.4–6.6) years. The median sMg level in the low sMg group was 1.7 (1.6–1.8) and 2.0 (2.0–2.2) mg/dL in the normal/high sMg group. The low sMg group was older (mean of 72 vs. 67 years), less likely to be male (64% vs. 72%), and had higher rates of comorbidities, including diabetes, hypertension, and atrial fibrillation (59% vs. 29%, 92% vs. 85%, and 6% vs. 5%; *p* < 0.05 for all). Kaplan–Meier survival analysis demonstrated significantly higher cumulative death probability at 4 years in the low sMg group (34% vs. 22%; *p* log rank <0.001). In a multivariable analysis model adjusted for sex, significant comorbidities, coronary interventions during the hospitalization, and renal function, the low sMg group exhibited an independent 24% increased risk of death during follow up (95% CI 1.11–1.39; *p* < 0.001). Conclusions: Low sMg is independently associated with higher risk of long-term mortality among patients recovering from an NSTEMI event.

## 1. Introduction

Following a first acute myocardial infarction (MI) episode, patients are confronted with a substantial risk of further cardiovascular events, including death, recurrent MI, heart failure, arrhythmias, angina, and stroke. Although there have been notable advancements in the medical care of patients with non-ST elevation myocardial infarction (NSTEMI), in particularly the widespread use of reperfusion techniques and the adjunctive use of multiple medical therapies, a substantial risk of cardiovascular events persists [1]. In contrast to the more favorable short-term prognosis, patients with NSTEMI experience similar or even higher long-term mortality rates when compared to those with ST-elevation MI [2,3]. Patients, as well as their family members, frequently seek insights into their future health trajectory. Therefore, prognostic information following an2 NSTEMI is imperative for comprehensive patient care.

Magnesium, the second most common intracellular cation, plays a critical role in many physiological processes, including cardiovascular function.

Some of the classical cardiovascular risk factors, including age, hypertension, and diabetes, are also associated with a low magnesium state [1,2,3], resulting in an apparent overlap between risk factors for cardiovascular disease and states associated with hypomagnesemia.

There are several possible mechanisms through which low serum magnesium (sMg) levels may worsen the course and outcome of patients with an acute cardiovascular event. Magnesium deficiency has been linked with the upregulation of interleukin-6, inhibiting endothelial proliferation and thereby contributing to the formation of atherosclerotic plaques and the activation of the coagulation cascade [4]. Low magnesium states are also associated with endothelial dysfunction through inhibition of endothelial release of nitric oxide, a vasodilator and inhibitor of platelet aggregation and adhesion [5]. Conversely, magnesium supplementation has been shown to improve brachial endothelial function in a randomized, double-blind, placebo-controlled trial of 50 stable coronary artery disease patients [6]. Magnesium’s antagonistic effect on calcium reduces calcium release from the sarcoplasmic reticulum, thereby protecting ischemic myocardial cells from calcium overload [7] as well as promoting vascular relaxation, reducing blood pressure and increasing coronary blood flow [8,9]. Furthermore, magnesium may have antiarrhythmic properties. Magnesium deficiency is associated with intracellular hypopotassemia, hypernatremia, and augmentation of cell excitability and has been associated in observational studies with higher rates of sudden cardiac death [10,11,12]. These effects may lead to ischemic myocardial damage and increased mortality in hypomagnesemic patients with acute coronary syndrome (ACS) [13].

Previous small studies have indicated a potential mitigating effect of magnesium supplementation on these adverse effects. A trial of 50 stable coronary artery disease (CAD) patients showed reduced ischemic ST-segment changes and improved exercise tolerance with magnesium therapy [6]. Additionally, an extended 30 year follow-up study established a link between dietary magnesium intake and a decreased likelihood of CAD [13].

The aim of our study is to increase our understanding of the role of sMg in the progression and outcomes of ACS by assessing the correlation between sMg levels upon admission and significant clinical outcomes in hospitalized NSTEMI patients.

## 2. Methods

### 2.1. Study Population

This is a retrospective cohort study of all adult patients admitted to the Sheba Medical Center between August 2008 and December 2019 with a diagnosis of NSTEMI who had sMg levels documented within three days (<72 h) of hospital admission. It is based on the previously described SHEBAHEART big data registry [14,15,16]. The Institutional Review Board of the Sheba Medical Center approved this study on the basis of strict maintenance of participants’ anonymity during database analyses. No individual consent was obtained.

Patients were included if NSTEMI was considered their primary diagnosis by the treating physician. The criteria for establishing this diagnosis in our institute during the study period were consistent with the universal definitions of myocardial infarction, based upon the Third and Fourth Universal Definition of Myocardial Infarction. sMg levels were measured using a photometric color test and are expressed as mg/dL (conversion factor from mg/dL to mmol/L, multiply by 0.4114).

**Figure 1:**
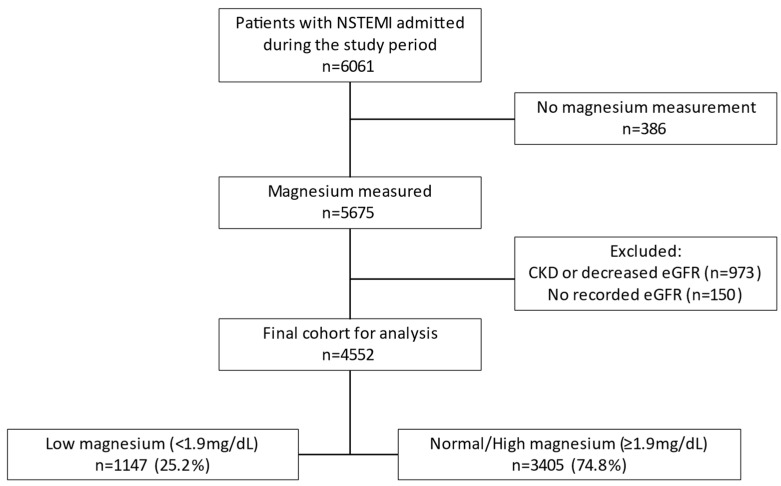
Flow chart of the study population.

Exclusion criteria included missing data (laboratory, pharmacologic, and/or invasive management) and advanced chronic kidney disease (Figure 1). sMg levels are increased in patients with renal failure secondary to a reduction of magnesium’s excretion in the urine [17]. Moreover, it has been shown that these elevations in sMg are not independently associated with poor outcomes but rather serve as a marker for severe renal dysfunction and its implications [18,19,20]. In our cohort, we witnessed an increase in sMg levels in patients with a creatinine clearance (CrCl) of less than 30 mL/min/1.73 m^2^, calculated by the CKD-EPI formula upon admission (Supplementary Figure S1). Therefore, and in order to avoid the confounding effect of renal failure, patients with known end-stage chronic kidney disease or CrCl of less than 30 mL/min/1.73 m^2^ upon admission were excluded.

Patients were divided into four mutually exclusive groups according to approximate sMg quartiles upon admission. We then dichotomized the study population into two groups: patients with low sMg levels (Q1; sMg < 1.9 mg/dL) vs. all other patients (Q2–Q4; sMg ≥ 1.9 mg/dL).

### 2.2. Study Variables and Outcomes

Patients’ baseline demographic and clinical data were retrieved from patients’ computerized records. Baseline, in-hospital, and discharge diagnoses were based on computerized hospitalization records (International Classification of Diseases, Ninth Revision codes), laboratory tests, medications, physiological signals (e.g., electrocardiograms [ECGs]), radiological images (e.g., echocardiograms, angiograms), and reports of procedures. The kit for troponin-I level measurements in our institute has changed throughout the study period, with a subsequent change in the units and reference range (from mcg/L to ng/L). In order to correct for that change, all troponin measurements were divided into quartiles and presented as such. The primary outcome was all-cause mortality, which was attained from the National Israeli Population Registry and was available for all patients.

### 2.3. Statistical Analysis

All the tests were two-tailed. *p* < 0.05 was considered significant. Continuous variables were presented as median (interquartile range) and categorical variables as *n* (%). Kaplan–Meier curves are shown. Cox proportional-hazard models were used to determine the hazard ratio between low serum magnesium and mortality, with normal/high sMg as the reference group. The proportional hazards assumption was visually confirmed. The adjusted model accounted for age, sex, smoking status, common comorbidities (atrial fibrillation, congestive heart failure, hypertension, ischemic heart disease, diabetes mellitus, and stroke), interventions during the hospitalization (catheterization and PCI), and CrCl.

The data were analyzed using R version 4.3.1 (R Core Team, Vienna, Austria) and packages tidyverse, survival, ggsurvfit, gtsummary, and patchwork.

## 3. Results

The final study cohort consisted of 4552 patients (70% male, median age 69 (59–79)). There were 1147 (25%) patients in the low sMg group and 3405 (75%) patients in the non-low group. Overall, the median follow-up of the study population was 4.4 (IQR 2.4–6.6) years.

Patients’ baseline characteristics are presented in Table 1. The median sMg level in the low sMg group was 1.7 (1.6–1.8) mg/dL and 2.0 (2.0–2.2) mg/dL in the normal/high sMg group. The distribution of sMg in the entire population, as well as in both groups, is graphically displayed in Supplementary Figure S2. The range of sMg was 0.7–1.88 mg/dL in the low sMg group and 1.9–3.2 mg/dL in the normal/high sMg group. 

Patients in the low sMg group were older (median of 72 years [IQR 63.5–81] vs. 67 years [IQR 58–79]; *p* < 0.001), less likely to be male (64% vs. 72%; *p* < 0.001), and had higher rates of comorbidities including diabetes, hypertension, atrial fibrillation, peripheral artery disease, and history of stroke (59% vs. 29%, 92% vs. 85%, 6% vs. 5%, 7% vs. 5%, and 8% vs. 6%, respectively; *p* < 0.05 for all). There was no significant difference between the groups in rates of congestive heart failure (4% in the low sMg group vs. 3% in the normal/high sMg group; *p* = 0.074), dementia (2% in both groups; *p* = 0.4), and smoking (31% in the low sMg group vs. 33% in the normal/high sMg group; *p* = 0.2).

There was no significant difference in the rate of conduction abnormalities such as left or right bundle branch blocks between the groups (4% in the low sMg group vs. 3% in the normal/high sMg group; *p* = 0.5 and 10% vs. 9%; *p* = 0.1, respectively). The laboratory data revealed lower hemoglobin and CrCl in the low sMg group (median of 11.7 g/dL [IQR 10.3–13.2] vs. 13 g/dL [IQR 11.4–14.2]; *p* < 0.001 and 69.5 mL/min/1.73 m^2^ [IQR 52.1–86.9] vs. 76.9 mL/min/1.73 m^2^ [IQR 57.9–92.6]; *p* < 0.001, respectively). Moreover, we witnessed higher maximal troponin in the low sMg group (highest quartile of troponin values at 33% in the low sMg group vs. 28% in the normal/high sMg group; *p* = 0.001) and higher glucose levels in the low sMg group (mean of 192 [IQR 145–271] vs. 146.2 [IQR 120–196]; *p* < 0.001). Echocardiographic data were available for 77% of the study cohort and demonstrated a similar left ventricular ejection fraction (LVEF) that was marginally lower in the low sMg group (median of 55% (IQR 40–60%) vs. 55% (IQR 45–60%); *p* < 0.001) and similar right ventricular function (right ventricular dysfunction of 5% in the low sMg group vs. 4% in the normal/high sMg group; *p* = 0.3).

Patients in the low sMg group were less likely to undergo coronary angiogram during their index hospitalization (62% vs. 70%; *p* < 0.001), and out of those who did, there was a higher rate of three-vessel disease (41% vs. 32%; *p* < 0.001) with a lower rate of percutaneous coronary interventions (39% vs. 46%; *p* < 0.001).

### 3.1. Subanalysis by Sex

Due to a higher rate of female patients in the low sMg group, we performed a subanalysis of the cohort by sex (Table 2).

In our cohort, sMg was similar in male and female patients: median sMg of 2.0 (IQR 1.9–2.1) mg/dL in the total male population, 1.7 (IQR 1.6–1.8) mg/dL in the male low sMg group and 2.0 (IQR 2.0–2.2) mg/dL in the male normal/high sMg group, *p* < 0.001; and median sMg of 2.0 (IQR 1.8–2.1) mg/dL in the total female population, 1.7 (IQR 1.6–1.8) mg/dL in the female low sMg group and 2.0 (IQR 2.0–2.2) mg/dL in the female normal/high sMg group, *p* < 0.001. Similarly to the main cohort, in both the male and female subpopulations, patients in the low sMg group were younger (69 years [IQR 61–78] vs. 65 years [IQR 56–75]; *p* < 0.001; and 78 years [IQR 70–84] vs. 74 years [IQR 64–84]; *p* < 0.001, respectively) and had higher rates of comorbidities including diabetes (61% vs. 29% and 56% vs. 29%, respectively), hypertension (91% vs. 84% and 93% vs. 88%, respectively), peripheral artery disease (8% vs. 5% and 7% vs. 5%, respectively), and history of stroke (8% vs. 5% and 9% vs. 7%, respectively; *p* < 0.05 for all). Moreover, the low sMg group exhibited lower hemoglobin (12.4 g/dL [IQR 10.8–13.7] vs. 13.5 g/dL [IQR 12.1–14.6] in the male subgroup; *p* < 0.001; and 10.9 g/dL [IQR 9.7–12.1] vs. 11.9 g/dL [IOR 10.6–12.8] in the female subgroup; *p* < 0.001), lower CrCl (73.2 mL/min/1.73 m^2^ [IQR 56.3–89.7] vs. 78.8 mL/min/1.73 m^2^ [IQR 60.8–93.9] in the male subgroup; *p* < 0.001; and 63.3 mL/min/1.73 m^2^ [IQR47.7–82] vs. 69.9 mL/min/1.73 m^2^ [IQR 51.7–88.8] in the female subgroup; *p* < 0.001) and higher maximal troponin (highest quartile of troponin values at 36% vs. 29% in the male subgroup; *p* < 0.001; and 26% vs. 24% in the female subgroup; *p* < 0.001). The LVEF was lower in the low sMg group (50% [IQR 40–60] vs. 55% [IQR 45–60] in the male subgroup; *p* < 0.001; and 55% [IQR 40–60] vs. 60% [IQR 45–60] in the female subgroup; *p* < 0.001).

### 3.2. Survival during Follow-Up

**Figure 2:**
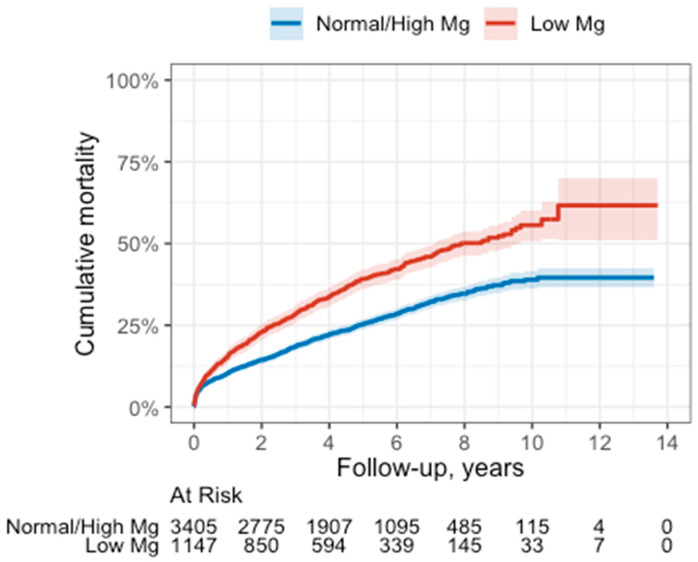
Survival curve in the low vs. normal/high sMg groups. Below the graph are the numbers of patients at risk.

During the follow-up period, 1416 (31%) patients died. There were 484 (42%) deaths in the low sMg group, compared with 932 (27%) in the non-low sMg group (*p* < 0.001). Kaplan–Meier survival analysis demonstrated that the cumulative probability of death at 4 years was 34% (95% CI 31–37%) among the low sMg group compared with 22% (95% CI 21–24%) among all other patients (*p* log rank < 0.001). Consistently, univariate Cox regression survival analysis demonstrated that compared with all other patients, low sMg patients were 64% more likely to die during follow-up (95% CI 1.47–1.83; *p* < 0.001) (Figure 2).

In a multivariable analysis model, adjusted for age, sex, smoking status, common comorbidities (including atrial fibrillation, congestive heart failure, hypertension, ischemic heart disease, diabetes mellitus, and stroke), interventions during the hospitalization (catheterization and PCI), and renal function, the low sMg group exhibited sustained elevated mortality rates (HR 1.24, 95% CI 1.11–1.39l; *p* < 0.001) (Table 3).

## 4. Discussion

The main finding of the current analysis shows that low sMg levels are associated with increased long-term risk of death in a large cohort of NSTEMI patients. The association was independent of other predictors of adverse outcome. To the best of our knowledge, this is the largest trial thus far to investigate the role of sMg in NSTEMI patients.

Mild hypomagnesemia is a common electrolyte abnormality [21]. Whether this abnormality should be treated or prevented with prophylactic magnesium administration is unclear. This matter assumes even greater importance in the context of atherosclerotic disease, as low sMg is associated with cardiovascular risk factors that may predispose to an acute ischemic event and potentially result in more extensive ischemic damage, leading to worse outcomes.

Furthermore, there is currently no international consensus on the normal sMg range [22]. Recently, an alternative threshold for defining low magnesium has been proposed [23]. We therefore divided our cohort into quartiles of sMg levels, rather than setting a particular threshold for either low or high magnesium, so that the low and high magnesium groups in our cohort represent a relative rather than an absolute finding. Interestingly, the low sMg group was found to have magnesium levels below the corresponding lower limit of normal in the assay used at our center (<1.9 mg/dL) [23]. Importantly, as only 1% of total body magnesium is in the serum, sMg measurement does not necessarily reflect its intracellular level. The most accurate intracellular magnesium measurements, which also reflect the intramyocardial muscle cell content, are lymphocytic (more accurate) and erythrocyte (less accurate and cell age-dependent) magnesium levels [24,25]. However, sMg remains the primary test for magnesium status in the absence of a more selective, reliable, and easily testable biomarker, and when low, it reliably reflects a total body magnesium depletion [22,26].

In our cohort, females were more likely to have lower sMg levels, consistent with previous reports [27]. However, the low sMg group in both sexes exhibited similar sMg levels, suggesting that while females are more likely to be magnesium depleted, the extent of depletion is similar in male patients. Furthermore, comparable patterns in baseline characteristics between the low and normal/high sMg groups were observed in both males and females.

Additionally, lower sMg levels are correlated with an increased prevalence of diabetes, as previously described in other populations, possibly due to changes in cellular glucose transport, insulin secretion, or insulin receptor binding [28].

We witnessed a higher rate of hypertension, atrial fibrillation, stroke, and peripheral arterial disease in the low sMg group. The association between atrial fibrillation and low magnesium levels, as well as its correlation with atherosclerosis, has been consistently reported [1,7,23,29,30,31,32] and may be mitigated by endothelial dysfunction, increased inflammatory activity, and calcium overload within the myocardial cells (see Introduction section) [4,5,6,7,8,33,34,35,36]. For instance, Amighi et al. [32] followed 323 patients with peripheral artery disease and intermittent claudication for 2 years and found that low sMg concentrations were associated with a 3-fold incidence of cerebrovascular accident compared to those with high sMg levels.

In terms of invasive therapy, patients in the low sMg group underwent coronary angiograms less frequently, and among those who did, the rate of a more advanced coronary disease was higher when fewer coronary interventions were performed. This observation may indicate an increased disease severity profile in this group, as evidenced by a higher comorbidity rate, reduced hemoglobin levels, worse renal function, and lower LVEF.

In our study, low magnesium was strongly associated with increased long-term mortality. This association remained significant even after including important risk factors in a multivariable analysis such as age, the presence of major comorbidities, renal function, and coronary intervention. Notably, most individuals in the low sMg group exhibited mild hypomagnesemia (median sMg of 1.7 (1.6–1.8) md/dL, see Supplementary Figure S2 for distribution of sMg levels across the cohort), underscoring the importance of strict maintenance of sMg levels within the normal range.

Previous studies have focused on the correlation between low sMg and short-term mortality in acute coronary syndrome patients, mainly due to the potential risk of arrhythmia and ischemia-reperfusion injury [37,38,39]. For example, a retrospective analysis of 259 acute MI patients from a single medical center revealed higher rates of arrhythmias, heart failure, cardiogenic shock, and death in patients with sMg levels of <1.82 mg/dL [4]. However, a randomized clinical trial (MAGIC) specifically designed to test the role of magnesium in the acute setting found no benefit [40]. We have therefore focused on the effect of sMg on long-term mortality.

Possible explanations for the increased mortality observed in the low sMg group in our study include the occurrence of ventricular arrhythmias late after the acute setting [41] and the more severe patient profile in this group, as indicated by the higher comorbidity rate, lower hemoglobin levels, worse renal function, and more advanced coronary disease. However, a multivariable analysis model was constructed to account for these factors, and it revealed sustained mortality rates in the low sMg group, even after adjusting for these variables. Therefore, we believe it is more likely related to long-term effects of low magnesium states such as increased infarct size, as evidenced by lower LVEF in the low sMg group and the heightened risk of recurrent cardiovascular events [37].

Indeed, large prospective epidemiologic studies have suggested that a low serum magnesium may be a risk factor for future coronary disease [30,31,42]. Moreover, in a Mendelian randomization study of over 180,000 individuals, a genetic predisposition to a higher serum magnesium level was associated with reduced risk of coronary artery disease [43]. The available data suggest that a combination of mechanisms may act additively or even synergistically in the formation of myocyte damage. Low magnesium state can impact vascular tone, platelet aggregation, the coagulation system, endothelial function, and infarct (scar) size and thus increase myocardial (re)infarction and heart failure rates. Due to its calcium antagonist effect and the fact it potentiates the dilatory action of some endogenous (adenosine, potassium, and some prostaglandins) and exogenous (isoproternol and nitroprusside) vasodilators, magnesium reduces systemic and pulmonary vascular resistance, with a concomitant decrease in blood pressure and afterload [7,26]. The antiplatelet effects of magnesium, mediated either by inhibiting platelet-stimulating factors, such as thromboxane A2, or by stimulating synthesis of platelet-inhibitory factors, such as prostacyclin, may prevent the propagation of coronary artery thrombi or re-occlusion of the infarct-related coronary artery after spontaneous or fibrinolysis-induced recanalization [44,45,46,47]. Moreover, low magnesium states are correlated with endothelial dysfunction, which, in turn, contribute to further platelet activation and vascular inflammation [5]. Magnesium deficiency may also adversely influence the healing and re-endothelialization of vascular injuries and increase vulnerability to oxygen-derived free radicals and may also result in delayed or inadequate angiogenesis. Such effects could potentially lead to inadequate collateral development and infarct expansion [48,49]. Lastly, magnesium deficiency has been associated with the increased expression of interleukin-6 and the initiation of an inflammatory response. This correlation holds paramount significance, as the immune system plays a pivotal role in the pathophysiology of cardiovascular disease [42,43,44,45].

These findings provide the pathophysiological foundation to the results of our study that low sMg could potentially serve as an early biomarker for long-term outcomes in NSTEMI patients.

## 5. Conclusions

Low sMg is an independent risk factor for long-term all-cause mortality in a large cohort of NSTEMI patients. Thus, low sMg levels upon admission may serve as an available, relatively inexpensive, and fast biomarker for poor outcomes and could aid in risk stratification of NSTEMI patients. Further prospective studies, focused specifically on long-term outcomes, are needed to establish a potential therapeutic role.

## 6. Strengths and Limitations

While this is the largest cohort of NSTEMI survival that investigated the prognostic significance of lower magnesium levels, we acknowledge several limitations. First, this was an observational study with a retrospective analysis of collected data. Second, the study was conducted in a single tertiary medical center, which may contribute to patient selection bias. Thus, the data cannot necessarily be extrapolated to other settings. Third, we evaluated the correlation between admission sMg levels and clinical outcomes, although intracellular magnesium levels are more accurate. However, these data were not available in this database. Additionally, certain parameters such as the NYHA score and TIMI flow score were unavailable. Nevertheless, our multivariable analysis, encompassing a comprehensive set of clinical parameters that reflect baseline comorbidity and clinical severity, revealed higher mortality rates in the low sMg group. Lastly, the immediate cause of death was unknown, which precludes making conclusions regarding magnesium levels and mechanism of death.

## Figures and Tables

**Table 1:** Patient characteristics and laboratory parameters by serum magnesium levels.

Characteristic	Low sMg (*n* = 1147)	Normal/High sMg (*n* = 3405)	Overall (*n* = 4552)	*p*-Value ^1^
Age	72.0 (63.5, 81.0)	67.0 (58.0, 79.0)	69.0 (59.0, 79.0)	<0.001
Male sex	736 (64%)	2439 (72%)	3175 (70%)	<0.001
Atrial fibrillation	74 (6%)	163 (5%)	237 (5%)	0.028
Ischemic heart disease	672 (59%)	2177 (64%)	2849 (63%)	0.001
Congestive heart failure	46 (4%)	100 (3%)	146 (3%)	0.074
Hypertension	1052 (92%)	2911 (85%)	3963 (87%)	<0.001
Diabetes mellitus	681 (59%)	982 (29%)	1663 (37%)	<0.001
Stroke	93 (8%)	189 (6%)	282 (6%)	0.002
Dementia	23 (2%)	84 (2%)	107 (2%)	0.4
Peripheral artery disease	85 (7%)	165 (5%)	250 (5%)	<0.001
Smoking	353 (31%)	1119 (33%)	1472 (32%)	0.2
LBBB	43 (4%)	114 (3%)	157 (3%)	0.5
RBBB	117 (10%)	293 (9%)	410 (9%)	0.10
Minimal hemoglobin—g/dL	11.7 (10.3, 13.2)	13.0 (11.4, 14.2)	12.7 (11.1, 14.0)	<0.001
Creatinine clearance—mL/min/1.73 m^2^	69.5 (53.1, 86.9)	76.9 (57.9, 92.6)	75.2 (56.5, 91.3)	<0.001
Magnesium—mg/dL	1.7 (1.6, 1.8)	2.0 (2.0, 2.2)	2.0 (1.9, 2.1)	<0.001
Maximal glucose—md/dL	192.0 (145.0, 271.5)	146.2 (120.0, 196.0)	155.0 (124.0, 215.0)	<0.001
Troponin by quartile Q1–3 Q4	738 (67%) 360 (33%)	2364 (72%) 903 (28%)	3102 (71%) 1263 (29%)	0.001
LVEF—%	55.0 (40.0, 60.0)	55.0 (45.0, 60.0)	55.0 (45.0, 60.0)	<0.001
RV systolic dysfunction	46 (5%)	118 (4%)	164 (5%)	0.3
Catheterization	710 (62%)	2380 (70%)	3090 (68%)	<0.001
PCI	446 (39%)	1561 (46%)	2007 (44%)	<0.001
Diseased coronary vessels 0 1 2 3	40 (7%) 137 (23%) 171 (28%) 246 (41%)	203 (10%) 694 (32%) 526 (25%) 682 (32%)	243 (9%) 831 (30%) 697 (25%) 928 (34%)	<0.001

Data are presented as *n* (%) or median (IQR), appropriately. ^1^ Pearson’s Chi-squared test; Wilcoxon rank sum test. LBBB = Left Bundle Branch Block; LVEF = Left Ventricular Ejection Fraction; PCI = Percutaneous Coronary Intervention; RBBB = Right Bundle Branch Block; RV = Right Ventricle; sMg = Serum Magnesium.

**Table 2:** Patient characteristics and laboratory parameters by sex and serum magnesium levels.

	Men			Women		
Characteristic	Low sMg	Normal/High sMg	*p*-Value ^1^	Low sMg	Normal/High sMg	*p*-Value ^1^
	(*n* = 736)	(*n* = 3405)		(*n* = 411)	(*n* = 966)	
Age	69.0 (61.0, 78.0)	65.0 (56.0, 75.0)	<0.001	78.0 (70.0, 84.0)	74.0 (64.0, 84.0)	<0.001
Hypertension	669 (91%)	2058 (84%)	<0.001	383 (93%)	853 (88%)	0.006
Diabetes mellitus	450 (61%)	703 (29%)	<0.001	231 (56%)	279 (29%)	<0.001
Stroke	56 (8%)	118 (5%)	0.004	37 (9%)	71 (7%)	0.3
Peripheral artery disease	58 (8%)	116 (5%)	0.001	27 (7%)	49 (5%)	0.3
Creatinine clearance—mL/min/1.73 m^2^	73.2 (56.3, 89.7)	78.8 (60.8, 93.9)	<0.001	63.3 (47.7, 82.0)	69.9 (51.7, 88.8)	<0.001
Minimal hemoglobin—g/dL	12.4 (10.8, 13.7)	13.5 (12.1, 14.6)	<0.001	10.9 (9.7, 12.1)	11.9 (10.6, 12.8)	<0.001
Troponin by quartile			<0.001			0.4
Q1–3	451 (64%)	1652 (71%)		287 (74%)	712 (76%)	
Q4	259 (36%)	681 (29%)		101 (26%)	222 (24%)	
LVEF—%	50.0 (40.0, 60.0)	55.0 (45.0, 60.0)	<0.001	55.0 (40.0, 60.0)	60.0 (45.0, 60.0)	0.008
RV systolic dysfunction	31 (5%)	87 (4%)	0.3	15 (5%)	31 (4%)	0.6
Catheterization	504 (68%)	1827 (75%)	<0.001	206 (50%)	553 (57%)	0.015
PCI	326 (44%)	1261 (52%)	<0.001	120 (29%)	300 (31%)	0.5
Diseased coronary vessels			<0.001			0.001
0	19 (4%)	91 (5%)		21 (13%)	112 (24%)	
1	99 (22%)	546 (33%)		38 (24%)	148 (31%)	
2	125 (28%)	418 (25%)		46 (29%)	108 (23%)	
3	191 (43%)	580 (35%)		55 (34%)	102 (22%)	

Data are presented as *n* (%) or median (IQR), appropriately. ^1^ Pearson’s Chi-squared test; Wilcoxon rank sum test. LBBB = Left Bundle Branch Block; LVEF = Left Ventricular Ejection Fraction; PCI = Percutaneous Coronary Intervention; RBBB = Right Bundle Branch Block; RV = Right Ventricle; sMg = Serum Magnesium.

**Table 3:** Multivariate analysis model of mortality rate compared with the normal/high serum magnesium group.

Characteristic	HR	95% CI	*p*-Value
Low serum magnesium levels	1.24	1.11, 1.39	<0.001
Age	1.07	1.06, 1.07	<0.001
Male sex	1.11	0.99, 1.24	0.079
Atrial fibrillation	1.01	0.84, 1.21	>0.9
Congestive heart failure	1.17	0.94, 1.44	0.2
Hypertension	1.18	0.97, 1.43	0.095
Ischemic heart disease	0.99	0.88, 1.11	0.8
Diabetes mellitus	1.25	1.12, 1.40	<0.001
Stroke	1.49	1.27, 1.76	<0.001
Smoking	1.37	1.20, 1.55	<0.001
Coronary angiography	0.45	0.39, 0.52	<0.001
Percutaneous coronary intervention	0.72	0.61, 0.85	<0.001
Creatinine clearance	0.99	0.98, 0.99	<0.001

CI = Confidence Interval; HR = Hazard Ratio.

## Data Availability

The data presented in this study are available on request from the corresponding author. The data are not publicly available due to patient privacy.

## Supplementary Materials

The following supporting information can be downloaded at: https://www.mdpi.com/article/10.3390/nu15194299/s1, Figure S1: Serum magnesium levels by kidney function. Figure S2: Serum magnesium levels histogram.
