# Supplementary material for: Serum Magnesium Is Associated with Long-Term Survival of Non-ST-Elevation Myocardial Infarction Patients
Source: Nutrients. 2023 Oct 9;15(19):4299. doi: 10.3390/nu15194299 (PMC10574643; doi:10.3390/nu15194299)

Supplementary Figure S1. Serum magnesium levels by kidney function.

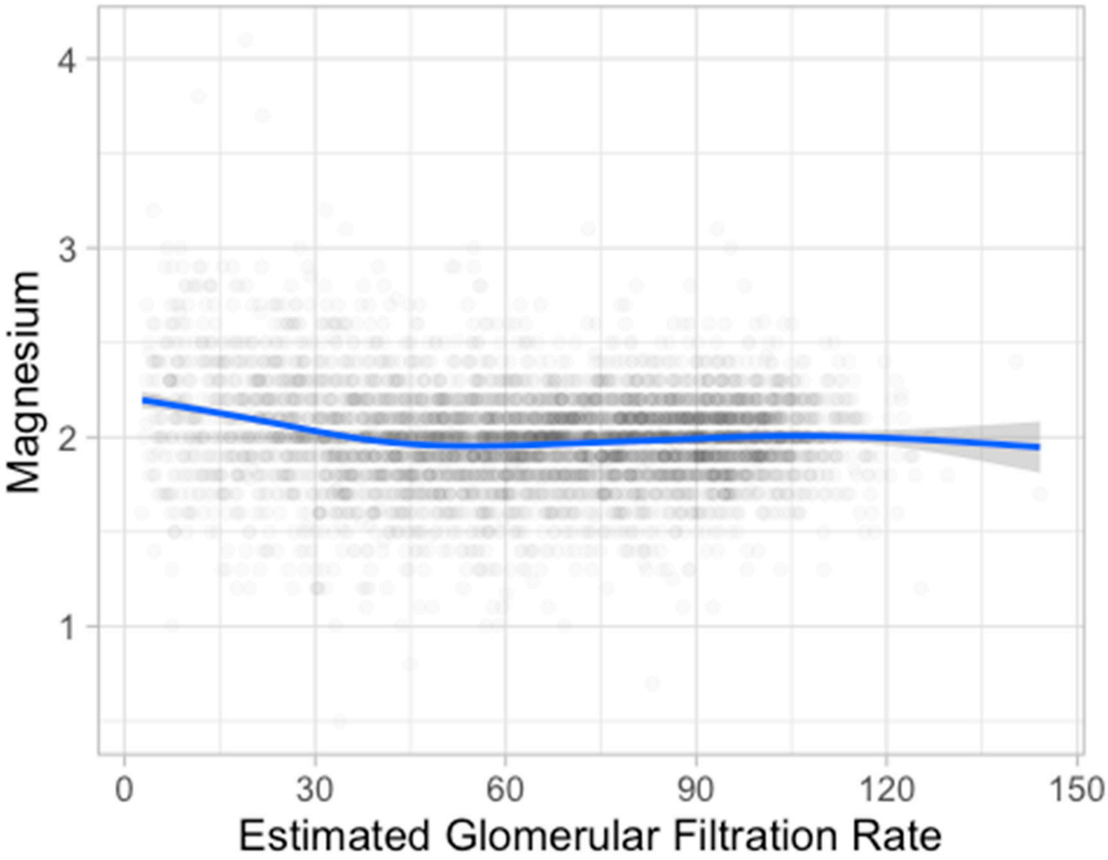

Supplementary Figure S2. Serum magnesium levels histogram

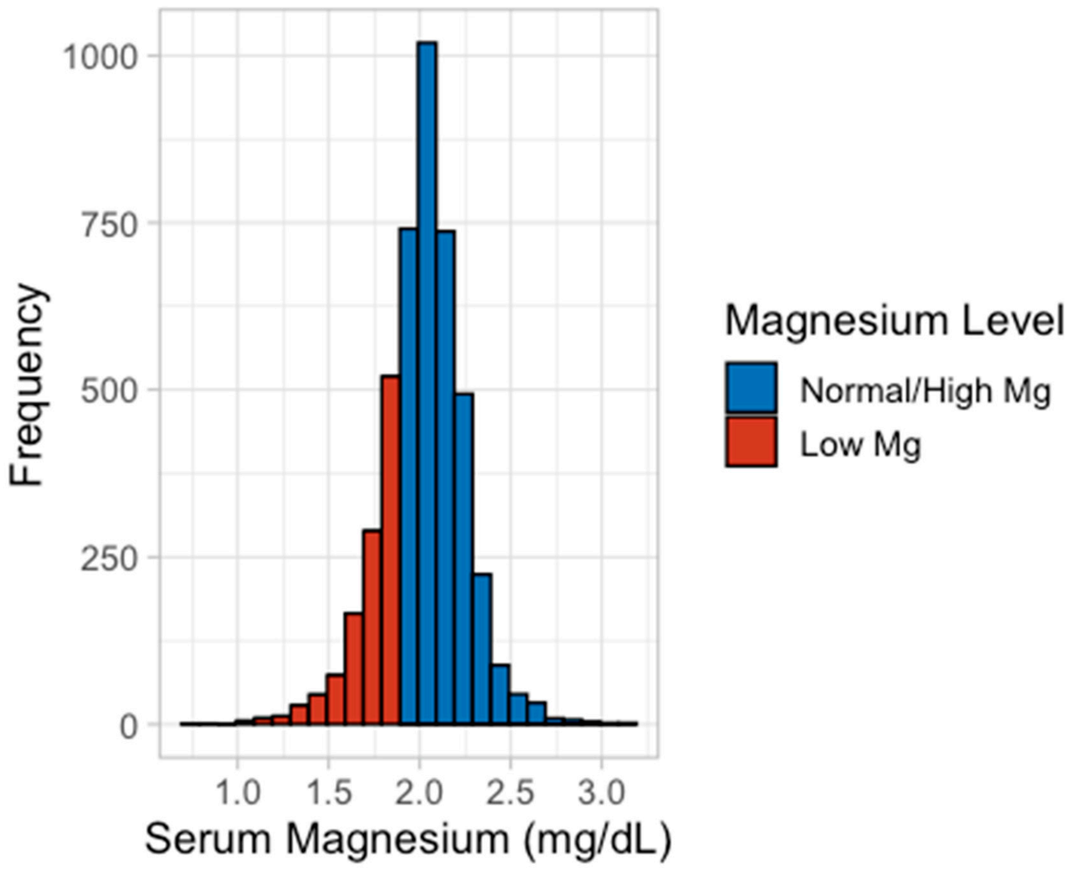

Supplement: Supplementary file 1 [file nutrients-15-04299-s001.zip › nutrients-2655317-supplementary.pdf]
